# Independent and Joint Impacts of Acid-Producing Diets and Depression on Physical Health among Breast Cancer Survivors

**DOI:** 10.3390/nu13072422

**Published:** 2021-07-15

**Authors:** K. Daniel Tessou, Hector Lemus, Fang-Chi Hsu, John Pierce, Suzi Hong, Lauren Brown, Tianying Wu

**Affiliations:** 1 Division of Epidemiology and Biostatistics, School of Public Health, San Diego State University, San Diego, CA 92182, USA; ktessou6831@sdsu.edu (K.D.T.); hlemus@sdsu.edu (H.L.); 2 Department of Biostatistics and Data Science, Division of Public Health Sciences, Wake Forest School of Medicine, Winston-Salem, NC 27101, USA; fhsu@wakehealth.edu; 3 Moores Cancer Center, School of Medicine, University of California, San Diego, CA 92037, USA; jppierce@ucsd.edu; 4 Department of Psychiatry, School of Medicine, University of California, San Diego, CA 92093, USA; s1hong@ucsd.edu; 5 Division of Health Management and Policy, School of Public Health, San Diego State University, San Diego, CA 92182, USA; lbrown2@sdsu.edu

**Keywords:** dietary acid load, depression, aging, physical health, breast cancer

## Abstract

The purpose of this study was to examine the independent and joint associations of acid-producing diets and depressive symptoms with physical health among breast cancer survivors. We studied a cohort of 2944 early stage breast cancer survivors who provided dietary, physical health, demographic, and lifestyle information at baseline, year 1, and year 4. We assessed the intakes of acid-producing diets via two commonly used dietary acid load scores: potential renal acid load (PRAL) and net endogenous acid production (NEAP). Physical health was measured using the Rand 36-Item Short Form Health Survey (SF-36), consisting of physical functioning, role limitation due to physical function, bodily pain, general health, and overall physical health subscales. Increased dietary acid load and depression were each independently and significantly associated with reduced physical health subscales and overall physical health. Further, dietary acid load and depression were jointly associated with worse physical health. For instance, depressed women with dietary acid load higher than median reported 2.75 times the risk (odds ratio = 2.75; 95% confidence interval: 2.18–3.47) of reduced physical function and 3.10 times the risk of poor physical health (odds ratio = 3.10; 95% confidence interval: 2.53–3.80) compared to non-depressed women with dietary acid load lower than median. Our results highlight the need of controlling acid-producing diets and the access of mental care for breast cancer survivors.

## 1. Introduction

As of 2020, breast cancer is the most diagnosed cancer among women in the United States [1]. The survival rate of breast cancer has increased drastically from 49% in 1975–1977 to 68% in 2004–2010 [1]; however, breast cancer survivors have accelerated aging and thus it is important to monitor and evaluate their health span and quality of life. Studying outcomes associated with aging will provide us insights of the aging process in breast cancer survivors. Research suggests among the domain of health-related quality of life, physical health may be the most important factor relating to cancer prognosis [2]. The Women’s Health Initiative-Observational Study examines health-related quality of life amongst women with a history of breast cancer and women without any history of cancer. The study found that breast cancer survivors had significantly lower physical function, more role limitations due to physical health, additional bodily pain, and poorer general health compared to women without a history of cancer [3]. Similar outcomes were observed in several other observational studies [4,5]. Physical function declines with age and has been found to predict aging-related outcomes such as reduced cognitive function, increased cardiovascular disease, and total mortality [6,7,8,9].

Investigating risk factors associated with physical health will provide informed prevention strategies for improving the precision of care among cancer survivors. Diet is one of the important behavioral risk factors. Several studies suggest a possible link between a healthy diet (low-fat and plant-based), which is known to produce low dietary acid and improved physical health quality of life among breast cancer survivors [10,11]. In today’s world, western diets consist of high acid-producing foods, such as unprocessed and processed meat, and low alkaline-producing foods, such as fruit and vegetables. Although some studies have reported that higher consumption of alkaline foods was associated with increased lifespan [12] and increased acid-producing diets were associated with increased mortality [13], results on alkaline diets and physical health are inconsistent. A randomized trial has shown that a vegetarian diet leads to greater improvement in physical and mental health among individuals with diabetes [14], whereas another cross-sectional study reported that a vegetarian diet was associated with poor mental health but no difference in physical health among vegetarians and semi-vegetarian women compared to non-vegetarians [15]. These discrepancies could be due to different study designs, different populations, and reverse causation in cross-sectional studies, as well as some other confounding factors. Moreover, they did not study the impact of acid-producing diets together with psychological factors, which may play important roles in physical health. To our knowledge, there are no prospective studies on the joint impact of acid-producing diets and mental health on physical health.

Psychological factors play an important role in physical health. The negative physical health impact of depression or the depression–physical illness associations have been documented [16]. Two studies exploring the relationship between depressive symptoms and physical health found a significant improvement in physical health after the administration of antidepressants and a positive correlation between improvement of physical health and reduction of depression [17,18]. These studies focused on the treatment of depression of physical health; however, to our knowledge, no longitudinal studies have examined the joint impacts of depression and acid-producing diets on physical health.

The current study utilized the Women’s Healthy Eating and Living (WHEL) data, which is a longitudinal study among breast cancer survivors. In this study, longitudinal data on dietary intake, depression, and physical health were analyzed. This study aimed to evaluate the independent and joint associations of acid-producing diets and depression with physical health among breast cancer survivors.

## 2. Materials and Methods

### 2.1. Study Design

The Women’s Healthy Eating and Living (WHEL) study is a multisite randomized control trial dating back between 1995 and 2000. The data related to cancer treatment, health status, physical function, and dietary intakes were collected at baseline, year 1, and year 4. The study enrolled 3088 women with the inclusion criteria of 18 to 70 years of age at diagnosis with operable invasive breast carcinoma at stage I (≥1 cm), stage II, or stage IIIA within the past 4 years. Only participants that were not undergoing chemotherapy, had no evidence of recurrence or metastasis, and had no other cancer within the past 10 years except nonmelanoma skin cancer or carcinoma in situ of the cervix were enrolled. Further details of the original WHEL study can be found in previous studies [13,19,20]. 

In this ancillary study, we also excluded participants who had missing data on depression, total calorie intake, and physical health assessments at baseline. After exclusion, 2944 participants were included in this study. The original study was approved by the University of California at San Diego Institutional Review Board (IRB), and written informed consent was obtained. Because we used de-identified data, the exempt IRB was approved by San Diego State University.

### 2.2. Dietary Assessment

Dietary consumption was accessed on four random days over 3 weeks by telephone. Two of the four days were on the weekend and two during the weekday. During the first year, participants were asked to recall a 24-h dietary intake. Trained dietary assessors used a multi-pass software-driven recall protocol of the Nutritional Data System software (NDS-R, 1994–2006, 91 University of Minnesota, Minneapolis, MN, USA).

On the basis of a 24-h recall, we obtained information of all the foods and drinks and the amount of each food and drink a participant consumed during the past 24 h. The NDS-R database calculated the amount of different nutrients in each food and drink. For instance, banana, spinach, and soymilk are enriched with magnesium; one medium banana contains 32 mg, ½ cup of boiled spinach contains 78 mg, and 1 cup of soymilk contains 61 mg of magnesium [21]. The total magnesium intake was calculated by summing the amount of magnesium in all the consumed foods and drinks in the past 24 h. These foods may include but not limited to nuts, soymilk, spinach, brown rice, and legumes. Because we have four 24-h recalls at each visit, the amount of magnesium at each visit was the average of magnesium intakes of the four 24-h recalls. Other nutrients were calculated in a similar way.

The dietary acid load was evaluated using the potential renal acid load (PRAL) and the net endogenous acid production (NEAP) score. The PRAL score estimates the production of endogenous acid that exceeds alkali produced for a given amount of food daily. The PRAL accounts for organic compounds and other materials (calcium, potassium, phosphorus, and magnesium) [22]. The NEAP score uses total protein and potassium consumed to derive dietary acid consumption [22]. A positive PRAL value signifies an increase in the production of acid precursors. A negative PRAL value indicates an increase in alkalinity [22]. The PRAL and NEAP scores were derived from the following:

$$\begin{array}{ll} PRAL\;\left(\frac{mEq}{day}\right) & =\left(0.49\times protein\;\left[\frac{g}{day}\right]\right)+\left(0.037\times phosphorus\;\left[\frac{mg}{day}\right]\right)-\left(0.021\times potassium\;\left[\frac{mg}{day}\right]\right)\\ & -\left(0.026\times magnesium\;\left[\frac{mg}{day}\right]\right)-\left(0.013\times calcium\;\left[\frac{mg}{day}\right]\right) \end{array}$$

$$NEAP\;\left(\frac{mEq}{day}\right)=\left(54.5\times protein\;\left[g/day\right]/potassium\;\left[mEq/day\right]\right)-10.2$$

### 2.3. Depression Assessment

Depressive symptoms assessment was based on the self-reported questionnaires using the 6-item short-form of the Center for Epidemiological Studies Depression Scale (CES-D). The CES-D has been acknowledged as a validated and reliable measure to evaluate symptoms of depression in breast cancer patients [23]. In the questionnaires, the following questions were used to evaluate depression: (1) “you felt depressed”, (2) “your sleep was restless”, (3) “you enjoyed life”, (4) “you had crying spells”, (5) “you felt sad”, and (6) “you felt that people disliked you”. The questions were rated on a three-point Likert scale: 0 = “none of the time”, 1 = “some of the time”, 2 = “a moderate amount of time”, and 3 = “most of the time”. Participants with a sum score ≥5 were classified as having elevated depressive symptoms [19].

### 2.4. Physical Health Assessment

The primary outcome of this study is physical health, which was measured using the Rand Short Form−36 survey (SF-36). The SF-36 consisted of the following subscales: physical functioning, role limitation due to physical function, bodily pain, general health, and overall physical health (summary of all four). All subscales of physical health were demonstrated to be reliable (Cronbach’s a = 0.75–0.91) and were validated [24]. A score of each subscale of physical health was calculated by transforming each item linearly to 0 (poor) to 100 (excellent) range scale, which was the percent of the total possible score; then, the average of all the items in the same scale was obtained to get the summary score for overall physical health score.

### 2.5. Other Assessment

The WHEL cohort had multiple clinical visits including baseline, year 1, and year 4 visits, and detailed health and demographic information were collected via questionnaires during these visits. Participants completed several questionnaires prior to the clinical visit that included self-reported Thoughts and Feelings Questionnaire, Personal Habits Questionnaire, Health Status Questionnaire, and Lifestyle Questionnaire. At the clinic visits, blood pressure, weight, height, and hip circumference were measured. Cancer-related information was retrieved from the participant’s medical records.

### 2.6. Statistical Analysis

Data analysis was conducted using SAS software version 9.4 (SAS Institute Inc., Cary, NC, USA). Univariate association of dietary acid load and depression was examined with the following variables at baseline: age at diagnosis, body mass index (BMI), ethnicity, menopausal stage, radiation therapy, chemotherapy, breast cancer stage, metabolic equivalents in minutes per week (METs), and smoking status. We used a *t*-test for continuous variables and chi-squared test for categorical variables. The dietary acid load was characterized as PRAL and NEAP scores. PRAL and NEAP scores were quartiled, and depression was treated as a binary variable.

The marginal model incorporating generalized estimating equations (GEEs) via GEMOD procedure in SAS was used to fit a generalized linear model assessing the association of dietary acid load and depression with outcome variables. This procedure estimates the parameters of the model through an iterative fitting process. It can handle repeated measures over time. The distributions of the outcome measures were examined and transformed if necessary in order to approximate the conditional normality assumption. The model was used to determine the longitudinal association between dietary acid load and depression with physical functioning, role limitation due to physical function, bodily pain, general health, and physical health. The exposure and outcome variables were time-vary variables at baseline, year one, and year four. Covariates were added in the model if they were confounders or predictive of the main outcome variables. We assessed potential confounders on the basis of the literature and confirmed them by correlation coefficient with main exposure and outcome variables. Both the age-adjusted model and multivariable-adjusted model included dietary acid load scores (PRAL/NEAP) and depression simultaneously. PRAL and NEAP were not adjusted simultaneously as they are highly correlated, and each evaluated dietary acid load from slightly different perspective. The multivariable model also included other covariates: physical activity quantified as METS age at diagnosis, menopausal status, BMI, estrogen and progesterone receptor status, smoking and pack per year status, total calorie intake, and comorbidities. Among these covariates, METS, BMI, and total calorie intakes are time-varying covariates. The *p*-value for linear trend for each dietary acid load score was assessed when acid load score was entered as a continuous variable; this continuous variable was created using the median of each quartile of PRAL or NEAP.

The joint impacts of dietary acid load (PRAL) and depression on outcome variables were analyzed using the marginal model with logit link and binomial distribution. Each outcome variable, such as physical function or bodily pain, was treated as a binary variable, and one of the independent variables (PRAL) was also transformed into a binary variable; all of these binary variables were created using the median of certain variable as the cut-off point. Women with CES-D score < 5 and low dietary acid load were treated as the reference group. The joint impacts of dietary acid load and depression on subscales of physical health were adjusted for METS, age at diagnosis, menopausal status, BMI, estrogen and progesterone receptor status, smoking and pack per year status, total calorie intake, and comorbidities.

## 3. Results

### 3.1. Baseline Descriptive Statistics

The baseline characteristics of 2944 women are displayed in Table 1. The study consisted mainly of white women (85.4%), women with diagnosis of breast cancer in middle age (mean = 50.8 ± 8.8), post-menopausal women (79.5%), and women who were not depressed (CES-D score < 5; 79.4%). Approximately 56.4% of women had stage I (*n* = 1659), 61.4% had radiation (*n* = 1808), and 69.7% had chemotherapy (*n* = 2052), and 60.5% drank alcohol (*n* = 2016).

### 3.2. Baseline Characteristics by Quartile of PRAL and Depression

Table 2 displays unadjusted univariate association of baseline characteristics with dietary acid load (PRAL) and depressive symptoms. We found that, compared to women with a lower PRAL score, women with higher PRAL score were more likely to be younger, obese, premenopausal, sedentary, have had chemotherapy, and less likely to be white. *p*-values were <0.05 for these comparisons. As shown in Table 2, compared to women reporting lower depressive symptoms, women with greater depressive symptoms were more likely to be younger and have normal BMI, they were less likely to be white, they were less likely to be in postmenopausal stage, and they were more likely to have metabolic equivalence between 0 and 600 min per week. These comparisons were statistically significant (*p*-value <0.05).

### 3.3. Mean SF-36 Physical Health Composite Score

**Figure 1:**
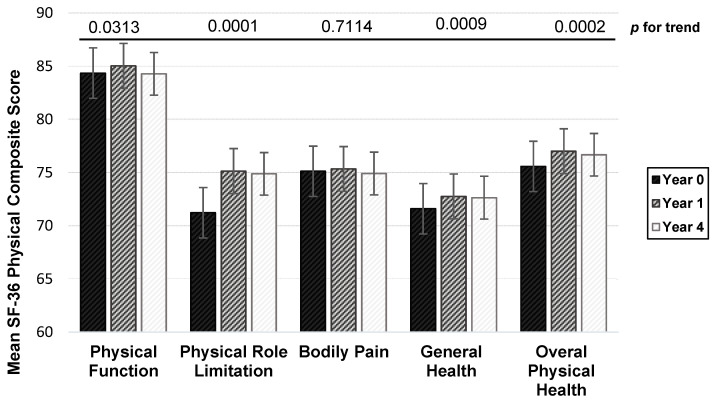
Mean summary score for each physical health measures at baseline, year 1, and year 4 among breast cancer survivors. Note: Overall physical health score is the average score of physical function, physical role in limitation, bodily pain, and general health. *p*-for-trend was obtained from the longitudinal generalized estimating equations model.

Figure 1 presents the mean scores of each physical health subscale and overall physical health over time. Physical function seemed to have improved among breast cancer survivors during the follow-up period. There were significant increases in mean scores for physical function, role limitation due to physical health, general health, and overall physical health (all *p*-values < 0.05). Among the physical health subscales, the greatest increase from baseline to year 4 was role limitation due to physical health (mean increased score = +3.65).

### 3.4. Independent Impact of Dietary Acid Load and Depression on Physical Health

Table 3 presents the age-adjusted and multivariable-adjusted association of dietary acid load and depression with different physical health measurements. In the age-adjusted models, PRAL, NEAP, and depression were significantly associated with physical function, role limitation due to physical health, bodily pain, general health, and overall physical health (*p*-for-trend < 0.001 for all these associations). In the age-adjusted analyses, compared to women with the lowest quartile of PRAL or NEAP, women with the highest quartile of PRAL or NEAP generally scored 5 to 8 points lower for each of the physical health measures.

In the multivariable model, the magnitudes of regression coefficients (beta estimates) were largely attenuated for PRAL and NEAP after adjustment of covariates. On the basis of *p*-values for trend, we found that PRAL continued to be significantly or marginally (*p*-value < 0.1) associated with most of the physical function measures, except for bodily pain; NEAP was significantly or marginally associated with all of physical health measures. Women with the highest quartile of PRAL and NEAP demonstrated lower physical function score (PRAL: β = −2.0, *p*-value = 0.001; NEAP: β = −2.08, *p*-value = 0.0015), compared to women with the lowest quartile of PRAL and NEAP.

The degrees of attenuations for the beta estimates between depression and physical health measures were much less in comparison to dietary acid scores, and depression remained significantly associated with all physical health measures. Compared to non-depressed women, women with depression displayed lower physical function (β = −6.77; *p*-value < 0.0001), more role limitation due to physical health (β = −18.4; *p*-value < 0.0001), increased bodily pain (β = −11.71; *p*-value < 0.0001), worse general health (β = −11.32; *p*-value < 0.0001), and poorer overall physical health (β = −12.07; *p*-value < 0.0001).

### 3.5. Joint Impact of Dietary Acid Load and Depression on Physical Health

In Table 4, each joint category of PRAL and depression was significantly associated with all of physical health measures when comparing to the reference group (*p*-value < 0.05). For instance, depressed women with dietary acid load higher than the median had 2.75 times the risk (odds ratio = 2.75; 95% confidence interval: 2.18–3.47) of physical dysfunction compared to non-depressed women with low dietary acid load. Depressed women with higher dietary acid load reported 3.10 times the risk of poor physical health (odds ratio = 3.10; 95% confidence interval: 2.53–3.80) compared to non-depressed women with low dietary acid load. The joint impacts of PRAL and depression on physical health were similar to that when PRAL was replaced by NEAP (data not shown).

### 3.6. Secondary Analyses: Independent Impacts of Dietary Acid Load and Depression on Physical Health Based on Age and Hormone Receptor Status

Table S1 shows the stratified analyses by age. The inverse associations between dietary acid load score (both PRAL and NEAP) and different subscales of physical health measures were still significant and even stronger in the strata with 47–55 years as compared to the whole data presented in Table 3; however, the associations were not significant and had no clear trends in other age strata. The inverse associations between depression and each physical health subscale were significant and similar to the whole dataset. Our results may indicate that the associations between dietary acid load score and physical health can be potentially modified by age, whereas the association between depression and physical health was not modified by age.

Furthermore, Table S2 shows the stratified analyses by hormone receptor status. Similar to the stratified analyses by age, depression was not modified by estrogen receptor (ER)+/progesterone receptor (PR)+ status and consistently and inversely associated with each physical health measure. For dietary acid load, the magnitudes of the inverse associations were stronger in ER+/PR+ strata; however, only NEAP maintained significant associations with most of the physical health measures.

## 4. Discussion

Overall, increased dietary acid load (PRAL/NEAP) and depressive symptoms were both independently associated with reduced overall physical health, and most of each physical health measures among breast cancer survivors, although the magnitude of these associations were stronger for depression. Further, our study demonstrated a joint impact of dietary acid load and depression on physical health: depressed women with higher dietary acid load had 2–3 times the risk of role limitation due to physical health, bodily pain, general health, and overall physical health than non-depressed women with lower dietary acid load.

Several longitudinal and cross-sectional studies have demonstrated that breast cancer survivors had a significantly worse physical health related quality of life compared to the general population of healthy women [3,5,25]. On the basis of our knowledge, studies on dietary acid load and physical function among breast cancer survivors are limited; however, some studies investigating the association between dietary pattern and physical functions may indirectly support our findings [11,26]. For instance, in a cross-sectional study among breast cancer survivors, women with western dietary pattern (high in meat, saturated fat, and total fat and low in fruit and vegetables) score at top 25% had −10 lower physical function score than women with western dietary pattern score at the bottom 25%; however, a causal relationship cannot be established due to its cross-sectional nature [11]. Further, their physical function score included emotion factor, physical role limitations, and general health problems. Nevertheless, western diets are acid-producing diets, and thus their findings are in agreement with ours.

Several mechanisms can help explain why acid-producing diets may lead to reduced physical health. Throughout the human body, countless chemical reactions are in equilibrium to maintain the body’s physical health. The equilibrium can be significantly affected by acid–base balance, which can be influenced by diet. Cancer survivors have a reduced capacity to adjust acid-base balance due to the toxicity of cancer treatment [27]. Acid-producing diets may potentially lead to metabolic acidosis [28] if the body has a reduced capacity to excrete acids. Metabolic acidosis lowers the serum levels of essential branched amino acids [29], which aids in muscle repair [30]; stimulates proteolysis [31], resulting in muscle degradation [32]; and exhausts endogenous levels of bicarbonate, which can neutralize acids. A cross-sectional study demonstrated that lower levels of bicarbonate were associated with loss of muscle mass and reduced physical function [33]; furthermore, a randomized controlled clinical trial reported that using bicarbonate as an alkaline treatment not only attenuated progression of muscle loss but also improved functional status and overall physical health [34].

Our examination of depression suggests that among breast cancer survivors, depression has a negative impact on all dimensions of physical health. Similar to our study, depression has been demonstrated to be associated with poorer physical health in other studies [35,36]. An cross-sectional observational study examining the impact of depressive symptoms on physical functioning reported a positive association between severity of depressive symptoms and worsening of physical functioning [35]. Some emerging theories have been proposed to explain the possible mechanisms. The harsh conditions incurred during cancer treatment (chemotherapy and radiation) has been correlated with the increase of inflammatory mediators such as C-reactive protein (CRP) and interleukin (IL)-6, which can promote depression [37]. Depression-associated dysregulation of the hypothalamic-pituitary-adrenal (HPA) axis, which plays crucial role in basal homeostasis, can further impact physical health [37]. Depression has been found to be accompanied by an upregulated inflammatory response [38,39]. This inflammatory response can stimulate hypothalamic and pituitary cells to release adrenocorticotropic hormone (ACTH) and cortisol, leading to glucocorticoid resistance and other adverse health outcome [38]. Glucocorticoids regulate a broad spectrum of physiologic functions, and thus disruption of glucocorticoids leads to various conditions such as chronic fatigue and physical dysfunction in the immune response leading to increased inflammation [38].

Our study has several strengths. The unique nature of our study means that it makes a novel contribution to the literature by being the first to examine the independent and joint associations of dietary acid load and depression symptoms with physical health among breast cancer survivors. In our study, depression and physical health were measured using well validated questionnaire. The original WHEL study obtained dietary information using multiple 24-h recalls, allowing us to better assess dietary intakes such as dietary acid load in comparison with a food frequency questionnaire. The large sample size allowed us to adjust for multiple covariates. We do acknowledge several limitations of this study. This study should not be generalized to all ethnic groups, as over 80% of our study participants were white women. The WHEL study had 6 years of follow-up, and thus we could not examine a longer-term impact of diet and depression on physical health beyond 6 years. Additionally, people who have worse physical health may also be depressed or be more likely to report depressive symptoms, and thus it is possible that depressive symptoms are both a cause and effect of poor physical health. Participants of the WHEL study were recruited on a volunteer basis, which could have caused a volunteer/selection bias. Finally, we did not collect some restricted diets such as ketogenic, vegetarian, and weight-loss diets, but our 24-h recalls did collect the exact intakes of all foods and drinks that participants consumed in two weekdays and two weekends. These collected dietary information are likely to capture an average level of these special diets but not all. Whether some special diets will confound the results will need to be investigated in the future.

## 5. Conclusions

In summary, a decline in physical function is one of the hallmarks of aging and is greatly impacted by emotional health and health behavior. Depression and acid-producing diets were significantly associated with reduced overall physical health and several types of physical function in breast cancer survivors. Currently, the American Cancer Society recommends healthy diets such as eating foods high in nutrients such as fruits and vegetables, as well as limiting processed products such as processed meats and refined grain products; however, there are no dietary guideline related to acid-producing diets [40]. Further, post-cancer treatment recommendations of mental healthcare for breast cancer survivors are urgently needed. It is important to set up an appropriate dietary acid load score for breast cancer survivors. More longitudinal and randomized trials to address the impact of acid-base balance and depression on physical health among breast cancer survivors are needed.

## Figures and Tables

**Table 1:** Baseline descriptive statistics.

Characteristics	Value (*n* = 2944)
Age, mean (SD)	50.8 (8.8)
Alcohol, *n* (%)	
Abstainer	928 (31.5)
Drinker	2016 (60.5)
BMI, *n* (%)	
Normal	1265 (43)
Overweight	907 (30.8)
Obese	772 (26.2)
Smoking Status, *n* (%)	
Current	133 (4.5)
Former	1224 (41.6)
Never	1587 (53.9)
Ethnicity, *n* (%)	
White	2515 (85.4)
Menopause, N (%)	
Premenopausal	327 (11.1)
Perimenopausal	274 (9.3)
Postmenopausal	2339 (79.5)
Depression, *n* (%)	
Depressed	608 (20.7)
Not depressed	2336 (79.4)
Radiation, *n* (%)	
Yes	1808 (61.4)
No	1132 (38.5)
Chemotherapy, *n* (%)	
Yes	2052 (69.7)
No	890 (30.2)
Stage, N (%)	
I	1138 (38.7)
II	1659 (56.4)
IIIA	147 (5.0)
METS, *n* (%)	
0–600	1468 (49.8)
600–1200	688 (23.4)
>1200	788 (26.8)

SD denotes standard deviation; BMI denotes body mass index; METS denotes metabolic equivalents in minutes per week.

**Table 2:** Baseline univariate associations of baseline characteristics with dietary acid load and depression among breast cancer survivors (*n* = 2944).

	PRAL Score (mEq/day)					Depression		
	Q1	Q2	Q3	Q4	*p*-Value	Depressed	Not Depressed	*p*-Value
Age at Dx, mean (SD)	52.1 (8.1)	52.4 (8.6)	50.8 (8.8)	48.8 (9.0)	<0.0001	49.5 (8.6)	51.1 (8.9)	<0.0001
BMI (kg/m^2^), *n* (%)					<0.0001			<0.0001
Normal	257 (59.9)	391 (50.2)	337 (38.4)	280 (32.6)		220 (36.2)	1045 (44.7)	
Overweight	116 (27.0)	230 (29.5)	316 (36.0)	245 (28.6)		181 (29.8)	726 (31.1)	
Obese	56 (13.1)	158 (20.3)	225 (25.6)	333 (38.8)		207 (34.1)	565 (24.2)	
Ethnicity, *n* (%)					<0.0001			0.0022
White, non-Hispanic	391 (91.1)	698 (89.6)	750 (85.4)	676 (78.8)		503 (82.7)	2012 (86.1)	
Menopause, *n* (%)					0.0001			0.0021
Premenopausal	31 (7.3)	67 (8.6)	98 (11.1)	131 (15.2)		67 (11.0)	260 (11.1)	
Perimenopausal	34 (8.0)	65 (8.4)	84 (9.5)	91 (10.6)		79 (13.0)	195 (8.4)	
Postmenopausal	360 (84.7)	642 (82.7)	700 (79.1)	637 (74.1)		460 (75.7)	1879 (80.4)	
Radiation, *n* (%)					0.4423			0.2758
Yes	265 (61.8)	493 (63.3)	519 (59.1)	531 (61.9)		380 (62.5)	1428 (61.1)	
No	164 (38.2)	286 (36.7)	357 (40.7)	325 (37.9)		226 (37.2)	906 (38.8)	
Chemotherapy, *n* (%)					0.0117			0.3829
Yes	283 (66.0)	512 (65.7)	631 (71.9)	626 (73.0)		436 (71.7)	1616 (69.2)	
No	146 (34.0)	266 (34.2)	246 (28.0)	232 (27.0)		172 (28.3)	718 (30.7)	
Stage, *n* (%)					0.5610			0.2306
I	169 (39.4)	297 (38.1)	332 (37.8)	340 (39.6)		251 (41.3)	887 (38.0)	
II	238 (55.5)	449 (57.6)	506 (57.6)	466 (54.3)		324 (53.3)	1335 (57.2)	
IIIA	22 (5.1)	33 (4.2)	40 (4.6)	52 (6.1)		33 (5.4)	114 (4.9)	
METS, *n* (%)					<0.0001			<0.0001
0–600	144 (33.6)	341 (43.8)	473 (53.9)	510 (59.4)		360 (59.2)	1108 (47.4)	
600–1200	114 (26.6)	206 (26.4)	206 (23.5)	162 (18.9)		134 (22.0)	554 (23.7)	
>1200	171 (39.9)	232 (29.8)	199 (22.7)	186 (21.7)		114 (18.8)	674 (28.9)	
Smoking status, *n* (%)					0.2777			0.1718
Former	193 (45.0)	325 (41.7)	360 (41.0)	346 (40.3)		259 (42.6)	965 (41.3)	
Current	10 (2.3)	37 (4.8)	41 (4.7)	45 (5.2)		35 (5.8)	98 (4.2)	
Never	226 (52.7)	417 (53.5)	477 (54.3)	467 (54.4)		314 (51.6)	1273 (54.5)	

PRAL denotes potential renal acid load; BMI denotes body mass index; METS denotes metabolic equivalents in minutes per week; SD denotes standard deviations; Q1, Q1, Q3, and Q4 denote quartile 1, quartile 2, quartile 3, and quartile 4, respectively.

**Table 3:** Impact of dietary acid load and depression on physical health subscales.

	PRAL Score (mEq/day)			NEAP Score (mEq/day)			Depression	
	Q1	Q4 β (*p*-Value)	*p*-Trend	Q1	Q4 β (*p*-Value)	*p*-Trend	Not Depressed	Depressed β (*p*-Value)
Physical function								
Age-adjusted	Ref	−7.79 (<0.0001)	<0.001	Ref	−7.78 (<0.0001)	<0.0001	Ref	−9.18 (<0.0001)
Multi-model	Ref	−2.00 (0.001)	0.01	Ref	−2.08 (0.0015)	0.0008	Ref	−6.77 (<0.0001)
Physical role limitation								
Age-adjusted	Ref	−6.57 (<0.0001)	<0.0001	Ref	−8.01 (<0.0001)	<0.0001	Ref	−21.09 (<0.0001)
Multi-model	Ref	−1.00 (0.44)	0.04	Ref	−1.94 (0.14)	0.03	Ref	−18.4 (<0.0001)
Bodily pain								
Age-adjusted	Ref	−5.18 (<0.0001)	<0.0001	Ref	−5.91 (<0.0001)	<0.0001	Ref	−13.78 (<0.0001)
Multi-model	Ref	−0.89 (0.28)	0.32	Ref	−1.37 (0.11)	0.002	Ref	−11.71 (<0.0001)
General health								
Age-adjusted	Ref	−4.90 (<0.0001)	<0.0001	Ref	−5.65 (<0.0001)	<0.0001	Ref	−13.35 (<0.0001)
Multi-model	Ref	−7.70 (0.30)	0.07	Ref	−1.24 (0.08)	0.09	Ref	−11.32 (<0.0001)
Overall physical health								
Age-adjusted	Ref	−6.09 (<0.0001)	<0.0001	Ref	−6.79 (<0.0001)	<0.0001	Ref	−14.37 (<0.0001)
Multi-model	Ref	−1.12 (0.10)	0.058	Ref	−1.56 (0.03)	0.005	Ref	−12.07 (<0.0001)

The multivariable-adjusted models were adjusted for PRAL/NEAP and depression simultaneously, METS, age at diagnosis, menopausal status, BMI, estrogen and progesterone receptor status, smoking and pack per year status, total calorie intake, and comorbidities. PRAL and NEAP were not adjusted simultaneously in the multivariable models. PRAL denotes potential renal acid load; BMI denotes body mass index; METS denotes metabolic equivalents in minutes per week; NEAP denotes net endogenous acid production; Q1 denotes quartile 1 and Q4 denotes quartile 4.

**Table 4:** Joint impact of dietary acid load and depression on physical health.

	PRAL Score (mEq/day)	
	<Median Odds Ratio (95% Confidence Interval)	≥Median OR (95% Confidence Interval)
Physical function		
Not depressed	Ref	1.28 (1.11–1.47) **
Depressed	2.36 (1.86–2.99) **	2.75 (2.18–3.47) **
Physical role limitation		
Not depressed	Ref	1.15 (1.00–1.31) *
Depressed	2.32 (1.86–2.91) **	2.47 (2.03–3.00) **
Bodily pain		
Not depressed	Ref	1.16 (1.03–1.31) *
Depressed	2.06 (1.66–2.55) **	2.56 (2.10–3.12) **
General health		
Not depressed	Ref	1.15 (1.02–1.30) *
Depressed	2.78 (2.21–3.50) **	3.30 (2.67–4.08) **
Overall physical health		
Not depressed	Ref	1.16 (1.02–1.31) *
Depressed	2.49 (1.99–3.11) **	3.10 (2.53–3.80) **

* *p*-value < 0.05, ** *p*-value < 0.001. The multivariable-adjusted models were adjusted for METS, age at diagnosis, menopausal status, BMI, estrogen and progesterone receptor status, smoking and pack per year status, total calorie intake, and comorbidities. PRAL denotes potential renal acid load; BMI denotes body mass index; METS denotes metabolic equivalents in minutes per week.

## Data Availability

Only dietary data and covariates are available online: https://library.ucsd.edu/dc/object/bb2493244b.

## Supplementary Materials

The following are available online at https://www.mdpi.com/article/10.3390/nu13072422/s1, Table S1: Impact of dietary acid load and depression on physical health subscales, stratified by different age strata, Table S2: Impact of dietary acid load and depression on physical health subscales, stratified by estrogen and progesterone receptor status.
