# Supplementary material for: Independent and Joint Impacts of Acid-Producing Diets and Depression on Physical Health among Breast Cancer Survivors
Source: Nutrients. 2021 Jul 15;13(7):2422. doi: 10.3390/nu13072422 (PMC8308757; doi:10.3390/nu13072422)
Supplement: Supplementary file 1 [file nutrients-13-02422-s001.zip › nutrients-1257499-supplementary.pdf]

Supplemental Table S1. Impact of dietary acid load and depression on physical health subscales, stratified by different age strata.

	PRAL Score (mEq/day)			NEAP Score (mEq/day)			Depression	
	Q1	Q4<br>β (p-value)	P- trend	Q1	Q4<br>β (p-value)	P- trend	Not Depressed	Depressed<br>β (p-value)
**<47 years old**								
Physical function	Ref	−1.52 (0.09)	0.2	Ref	−1.69 (0.09)	0.16	Ref	−6.19 (<0.0001)
Physical Role-limitation	Ref	0.52 (0.81)	0.38	Ref	1.11 (0.62)	0.08	Ref	−20.68 (<0.0001)
Bodily Pain	Ref	0.35 (0.81)	0.87	Ref	−0.08 (0.96)	0.32	Ref	−11.55 (<0.0001)
General Health	Ref	0.07 (0.95)	0.02	Ref	−0.70 (0.58)	0.04	Ref	−12.06 (<0.0001)
Overall Physical Health	Ref	−0.09 (0.94)	0.32	Ref	−0.29 (0.81)	0.07	Ref	−12.60 (<0.0001)
**47 – 55 years old**								
Physical function	Ref	−1.69 (0.08)	0.37	Ref	−1.77 (0.08)	0.06	Ref	−7.59 (<0.0001)
Physical Role-limitation	Ref	−2.66 (0.20)	0.53	Ref	−3.73 (0.08)	0.37	Ref	−17.98 (<0.0001)
Bodily Pain	Ref	−3.21 (0.01)	0.04	Ref	−3.42 (0.01)	0.001	Ref	−11.84 (<0.0001)
General Health	Ref	−2.60 (0.02)	0.13	Ref	−2.99 (0.008)	0.03	Ref	−11.58 (<0.0001)
Overall Physical Health	Ref	−2.54 (0.02)	0.12	Ref	−2.98 (0.008)	0.02	Ref	−12.24 (<0.0001)
**>55 years old**								
Physical function	Ref	−1.15 (0.40)	0.81	Ref	−0.82 (0.57)	0.39	Ref	−6.43 (<0.0001)
Physical Role-limitation	Ref	1.25 (0.62)	0.18	Ref	−1.49 (0.56)	0.18	Ref	−16.30 (<0.0001)
Bodily Pain	Ref	1.55 (0.34)	0.80	Ref	0.32 (0.85)	0.40	Ref	−11.38 (<0.0001)
General Health	Ref	0.78 (0.54)	0.44	Ref	0.20 (0.88)	0.49	Ref	−9.75 (<0.0001)
Overall Physical Health	Ref	0.68 (0.62)	0.57	Ref	−0.26 (0.86)	0.32	Ref	−11.05 (<0.0001)

The multivariable-adjusted models were adjusted for PRAL/NEAP and depression simultaneously, METS, age at diagnosis, menopausal status, BMI, estrogen and progesterone receptor status, smoking and pack per year status, total calorie intake, and comorbidities. PRAL and NEAP were not adjusted simultaneously in the multivariable models. PRAL denotes potential renal acid load; BMI denotes body mass index; METS denotes metabolic equivalents in minutes per week; NEAP denotes net endogenous acid production.

Supplemental Table S2. Impact of dietary acid load and depression on physical health subscales, stratified by estrogen and progesterone receptor status.

	PRAL Score (mEq/day)			NEAP Score (mEq/day)			Depression	
	Q1	Q4<br>β (p-value)	P- trend	Q1	Q4<br>β (p-value)	P- trend	Not Depressed	Depressed<br>β (p-value)
**ER+/PR+**								
Physical function	Ref	−1.98 (0.01)	0.09	Ref	−1.74 (0.04)	0.05	Ref	−6.57 (<0.0001)
Physical Role-limitation	Ref	−1.80 (0.28)	0.21	Ref	−2.60 (0.13)	0.13	Ref	−17.31 (<0.0001)
Bodily Pain	Ref	−1.03 (0.35)	0.30	Ref	−1.08 (0.34)	0.004	Ref	−11.08 (<0.0001)
General Health	Ref	−1.12 (0.20)	0.39	Ref	−1.76 (0.05)	0.23	Ref	−10.37 (<0.0001)
Overall Physical Health	Ref	−1.43 (0.11)	0.20	Ref	−1.69 (0.07)	0.04	Ref	−11.36 (<0.0001)
**The group without ER+/PR+**								
Physical function	Ref	−1.89 (0.06)	0.24	Ref	−2.17 (0.04)	0.03	Ref	−7.03 (<0.0001)
Physical Role-limitation	Ref	0.31 (0.88)	0.08	Ref	−0.97 (0.64)	0.18	Ref	−20.18 (<0.0001)
Bodily Pain	Ref	−0.65 (0.61)	0.76	Ref	−1.92 (0.15)	0.25	Ref	−12.64 (<0.0001)
General Health	Ref	−0.04 (0.97)	0.004	Ref	−0.48 (0.67)	0.11	Ref	−12.93 (<0.0001)
Overall Physical Health	Ref	−0.57 (0.59)	0.10	Ref	−1.38 (0.21)	0.10	Ref	−21.09 (<0.0001)

The multivariable-adjusted models were adjusted for PRAL/NEAP and depression simultaneously, METS, age at diagnosis, menopausal status, BMI, estrogen and progesterone receptor status, smoking and pack per year status, total calorie intake, and comorbidities. PRAL and NEAP were not adjusted simultaneously in the multivariable models. PRAL denotes potential renal acid load; BMI denotes body mass index; METS denotes metabolic equivalents in minutes per week; NEAP denotes net endogenous acid production.

The group without ER+/PR+ include women with EP+/PR-, ER-/PR+, ER-/PR- and unknow status.
